# Instrumented Crutch Tip for Monitoring Force and Crutch Pitch Angle

**DOI:** 10.3390/s19132944

**Published:** 2019-07-04

**Authors:** Iñigo Sesar, Asier Zubizarreta, Itziar Cabanes, Eva Portillo, Jon Torres-Unda, Ana Rodriguez-Larrad

**Affiliations:** 1 Department of Systems Engineering and Automation, University of the Basque Country UPV/EHU, 48013 Bilbao, Spain; 2 Department of Physiology, University of the Basque Country UPV/EHU, 48940 Leioa, Spain

**Keywords:** crutch, cane, wearable sensors, gait analysis, monitoring, rehabilitation

## Abstract

In rehabilitation procedures related to the lower limbs, gait monitoring is an important source of information for the therapist. However, many of the approaches proposed in the literature require the use of uncomfortable and invasive devices. In this work, an instrumented tip is developed and detailed, which can be connected to any crutch. The instrumented tip provides objective data of the crutch motion, which, combined with patient movement data, might be used to monitor the daily activities or assess the recovery status of the patient. For that purpose, the tip integrates a two-axis inclinometer, a tri-axial gyroscope, and a force sensor to measure the force exerted on the crutch. In addition, a novel algorithm to estimate the pitch angle of the crutch is developed. The proposed approach is tested experimentally, obtaining acceptable accuracies and demonstrating the validity of the proposed lightweight, portable solution for gait monitoring.

## 1. Introduction

Gait monitoring and analysis are of high interest, as patients suffering from a lower-limb impairment usually report not only a decrease in their autonomy and quality of life, but also a poorer health status [1,2,3]. Assessment of patient recovery in rehabilitation is usually carried out using various standardized tests and scales, which provide limited performance parameters or are subjective and coarse [4,5,6,7]. However, researchers have shown the benefits of using diagnosis instruments such as miniature sensors and optical systems for monitoring and assessing patient recovery [8,9,10,11].

Instrumented gait monitoring is usually performed using portable pressure-sensitive walkways, motion capture systems (MCS), or wearable sensors [12]. The first alternative is easy to use and accurate [13], but it does not provide information about the orientation of the body segments. The second approach provides accurate monitoring of all kinds of movements during gait, with instantaneous position errors under 1 mm [11], but their use is limited to indoor scenarios; their cost is high; and they require long set up and post-processing times [14,15]. On the other hand, wearable sensors provide a lightweight and minimally-invasive solution for different environments and long monitoring times with a much lower cost [8,15,16]. However, the attachment of the sensors to the body (including their location and orientation), the vibrations and noise, the drift or accumulated error, the external magnetic perturbations, and the complicated algorithms to estimate the orientation still present significant challenges [15,17,18].

In order to overcome these issues, some researchers have proposed the introduction of small motion sensors in passive rehabilitation devices such as crutches, so that the motion of the crutch can be used to monitor several variables [7,14,19,20]. For instance, in [21,22,23,24], instrumented crutches were used to monitor objectively and control loads applied on lower limbs of patients in rehabilitation. In addition, gait monitoring and biofeedback approaches can ensure a proper use of crutches during recovery [7,20,22,25,26]. Another application area is monitoring of the physical activity of the patient [21,22], as it is related to the health and recovery status [27,28,29]. Finally, instrumented crutches can also be used to perform clinical assessments based on the information provided by quantitative parameters [22], to estimate the risk of falls [30], or to detect other gait problems [31]. Hence, note that even if instrumented crutches cannot provide accurate information about the user’s gait kinematics such as the stance phase duration for each leg, the crutch motion and use is an important source of information for the therapists.

In order to fulfill these tasks, it is useful to measure two variables. First is the axial load force [23], which allows determining the support requirements of the patient. Second is the pitch angle of the crutch, as it allows defining the motion of the crutch [32], and it is useful for identifying gait performance indicators such as crutch dragging and imperfect alignment [22]. While the first variable is usually measured by piezoelectric sensors or load cells, the measurement of the crutch pitch angle is a more challenging task, for which gyroscopes or accelerometers are traditionally used.

The use of gyroscopes to measure crutch inclination requires integration of the measured angular rate, which introduces a drift [12,19,32,33] that has to be corrected. For that purpose, several approaches have been proposed, such as the use of pressure sensors to detect a reference position [19] or compensating the drift by estimating its tendency [32]. However, these approaches, in general, do not provide sufficient accuracy.

The use of accelerometers allows employing the gravity vector to determine the overall orientation of the crutch, as proposed in [17,22,27]. This approach is based on the assumption that patients using crutches walk slowly and that the module of the acceleration, which is obtained after subtracting the acceleration due to gravity, is negligible. This acceleration will be referred to as “nongravitational acceleration” in this work. However, assuming that nongravitational accelerations are negligible when the patient is moving slowly is only valid if the accelerometer is attached to the trunk of the body [34], as accelerations measured in the tip of the crutch are much larger.

In this work, a novel instrumented crutch tip for gait monitoring is presented. The proposed device is an adaptable instrumented tip that can be easily attached to any regular crutch or cane, which allows reducing the cost of the overall device and provides a lightweight and flexible solution. The crutch tip allows measuring exerted loads by means of a force sensor and inclination of the crutch by means of a novel processing approach based on a gyroscope, an inclinometer, and the detection of the different crutch phases using the force sensor. In contrast to other approaches in the literature, in order to demonstrate the validity of the inclination estimation approach, a dynamic validation is presented using a motion capture system.

The rest of the paper is structured as follows: in Section 2, a description of the proposed instrumented crutch tip is presented, together with the calibration of the force sensor, gyroscope, and inclinometer; in Section 3, the proposed algorithm for measuring crutch inclination is explained; Section 4 details the algorithm validation performed and presents the results; Section 5 discusses the results, comparing them with other approaches in the literature, and describes future work; and finally, in Section 6, the most important ideas and their relevance are summarized.

## 2. Instrumented Crutch Tip Prototype

**Figure 1:**
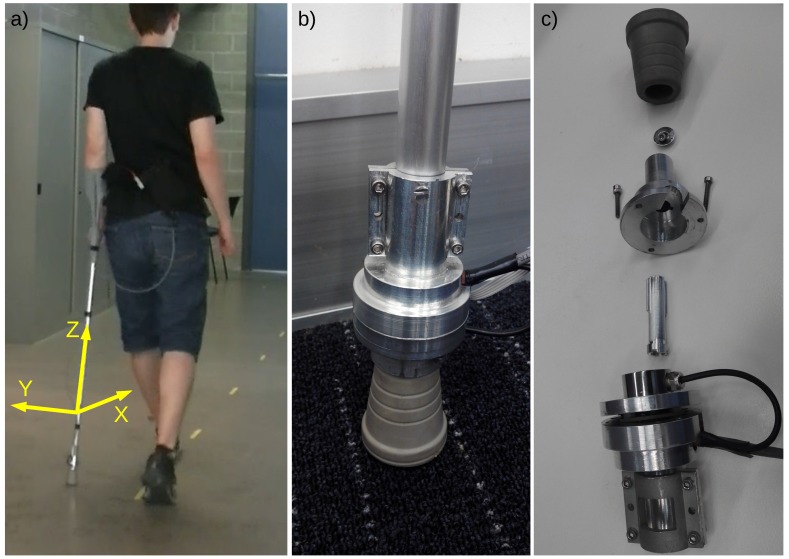
(**a**) A person using the instrumented crutch; (**b**) Assembled prototype tip; (**c**) Assembling the system.

The proposed instrumented crutch is shown in Figure 1. The custom-designed crutch tip was built in aluminum, and it was designed to include the required sensors and signal conditioning PCB. The tip was designed to fit different crutch diameters, so that it can be easily mounted on a regular crutch or cane and fixed using screws. In addition, the system requires a belt, which is fastened to the waist of the user and which includes the processing unit and the battery to power the instrumentation. Figure 1a shows a picture of a person using a regular crutch with the proposed instrumented tip.

The axial forces of the crutch were measured with an HBM C9C force sensor (1-kN nominal force), whose signal was then amplified using an INA118 IC (Texas Instruments). Crutch tilt angles were measured using the SCA100T-D02 dual axis inclinometer and the AltIMU-10 v5, which consisted of a 3-axis accelerometer, a 3-axis gyroscope, a 3-axis magnetometer, and a barometer (ST Microelectronics). The tilt angle estimation algorithm and the need for the aforementioned sensors are detailed in the next sections.

The processing unit was composed of a myRIO device, which was programmed in Labview RT. The designed data acquisition program consisted of a remote user interface with a control loop and a timed DAQ loop. The control loop executed every 100 ms and consisted of a state machine with three states (initialization, waiting, and capturing). The frequency of the data acquisition loop can be set by the user to any value between 10 and 100 Hz, as many instrumented crutches also acquire data at a frequency in this range [21,22,23]. This device captured raw data from the sensors and stored them in an LVMfile, which can then be processed to calculate different indicators such as mean speed, number of crutch movement cycles, mean of crutch inclination values at force peaks and percentage of body weight (PBW) applied on the crutch.

### 2.1. Calibration of Force Measurement

The force measurement was taken by an HBM C9C piezoelectric force sensor that was connected to a sliding aluminum part, which transmitted the axial force from the rubber tip of the crutch to the force sensor (Figure 1c). The load cell was isolated from off-axis loads and torques by preventing the rotation of the sliding part with two rails. The friction between the sliding part and the fixed housing, the deformation of the rubber tip, and the dynamics of the amplifier used to condition the voltage output of the sensor created nonlinearities in the force measurement. Due to these nonlinearities, a dead zone existed for small forces. In order to minimize this dead zone, a pre-load of about 10 N was applied before calibrating the force measurement.

An already validated, the Bertec 4060-15 force plate was used to calibrate the axial forces measured by the prototype. The rated load of the used force plate was 20 kN, and its maximum error due to nonlinearity and hysteresis was 0.2% of the rated load (40 N). The force plate recorded measurements at a frequency of 1000 Hz, but the signal was resampled at 100 Hz to compare it with the force-signal captured by the instrumented crutch. The calibration procedure consisted of positioning the crutch vertically on the force plate, with its rubber tip in contact with it, and applying different load/unload cycles on it. Random forces were applied to cover the whole range from 0–500 N and to consider the hysteresis effect. This procedure was repeated twice for 30 s, so that the first dataset was used to perform the calibration and the second to validate it. The measurements of the crutch DAQ system were synchronized with the data from the force plate by a fast impact of the crutch against the force plate at the beginning of each experiment.

**Figure 2:**
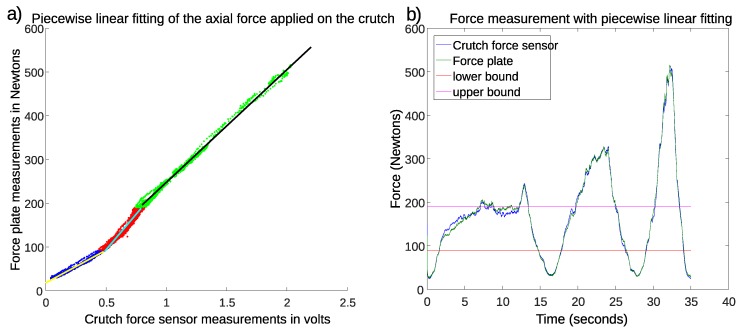
Results with force data of the first recording. (**a**) Best-fitting straight lines for each cloud. (**b**) Evolution of force measurements after calibration.

The data obtained in the first set are shown in Figure 2a. This figure represents the force plate measurement corresponding to each measurement of the force sensor integrated in the crutch. Note that, in this point cloud, the first 100 samples around the initial synchronization impact and the samples in the dead zone were not considered. It was observed that the force sensor was not able to measure voltages below 0.04 volts, and the force plate minimum force measurement was set to 25 N. The measurement noise of the raw force sensor signal was below 0.005 volts. In addition, the effect of the previously-detailed friction can be observed in low force ranges (below 200 N), where the fitting between the crutch force sensor and the force plate measurements is not linear. For this reason and in order to minimize the measurement errors, three piecewise linear approximations were calculated, as seen in Figure 2a: below 90 N (blue), between 90 and 190 N (red), and above 190 N (green). The best fitting straight line was calculated by least squares regression for each cloud in the selected ranges, obtaining the following results:

(1) $$\begin{array}{cc} y=149.102\;x+17.7974 & \;R^{2}=0.9752 \end{array}$$

(2) $$\begin{array}{cc} y=325.658\;x-69.3642 & \;R^{2}=0.9148 \end{array}$$

(3) $$\begin{array}{cc} y=257.714\;x-10.2081 & \;R^{2}=0.9956 \end{array}$$

The result of the aforementioned piecewise linear fitting for the first set (calibration set) is depicted in Figure 2b. Note that the two horizontal lines define the bounds separating the three linear intervals. It can be seen that there is no steep transition around the limits.

**Figure 3:**
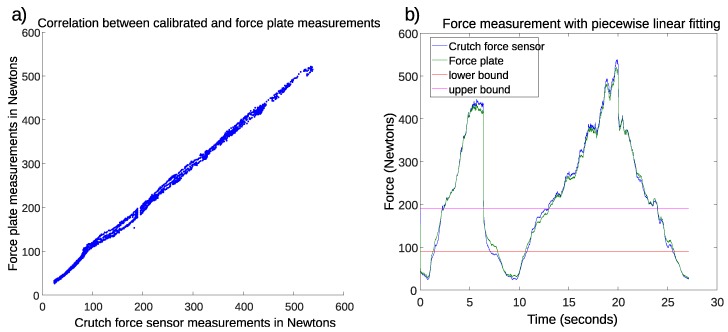
Results with data of the second recording after applying piecewise linear transformation. (**a**) Correlation between force plate and crutch force sensor measurements; (**b**) Evolution of force measurements.

The second dataset was used to verify the precision of the aforementioned calibration. Subsequently, the measurement pairs corresponding to the dead zone, synchronization impact, and fast load/unload intervals were discarded. Then, the piecewise linear transformation calculated with the first dataset was applied to the second dataset captured by the crutch sensors. Figure 3a shows the scatter plot that represents the correlation between the calibrated force measurements of the crutch and the reference force plate measurements. The coefficient of determination of the new cloud was $R^{2}=0.9957$. It can be seen that, unlike in Figure 2a, the cloud in Figure 3a showed a linear relationship between the force plate measurements and the calibrated crutch force sensor measurements in the whole measurement range, due to the calibration parameters calculated with the first dataset.

Figure 3b shows the time evolution of both the force plate measurement, used as a reference, and the calibrated crutch force sensor measurement for the second dataset. The horizontal lines determine the bounds separating the three linear intervals. The RMS error was 8.78 N, and the maximum absolute error was 26.84 N. The biggest errors occurred when measuring forces over 500 N. Note that these numbers included errors due to resolution, nonlinearity, and hysteresis.

**Figure 4:**
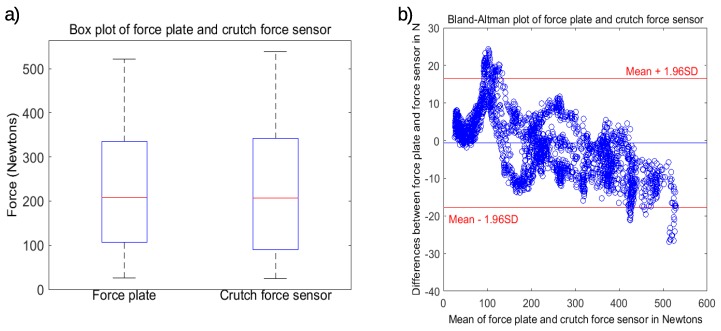
Results with data of the second recording after calibration. (**a**) Box plot. (**b**) Bland–Altman plot.

Figure 4a shows the box plots of the two signals represented in Figure 3b. The difference between the mean of the force plate measurements and the mean of the crutch force sensor readings was −0.64 N. Figure 4b presents the Bland–Altman plot of the same signals. The limits of agreement were 16.53 N and −17.8 N. The trend indicated that for higher loads, the crutch force sensor measurements were slightly larger than the force plate measurements, while they were smaller for lower loads. This plot also shows the most non-tolerable measurements group around the transition between the two lower lines of the linear fitting. The accuracy could be improved by increasing the number of subclouds and best-fitting lines.

Hence, these results show that the calibration of the force measurement was acceptable. For example, following a similar procedure, the crutches validated in [23] had a global coefficient of determination of 0.993, while the value obtained for our instrumented crutch was 0.9957. In addition, the RMS error obtained for the crutch in [22] was 9 N, while the RMS error calculated for our crutch with the data of the second recording was 8.78 N. This accuracy could be improved if the internal friction between the crutch tip parts was decreased.

### 2.2. Calibration of Inclination Measurement

In order to determine the crutch inclination measurement, a high-resolution and low-noise inclinometer and a triaxial gyroscope were used. In this section, the calibration procedure for both sensors is detailed.

**Figure 5:**
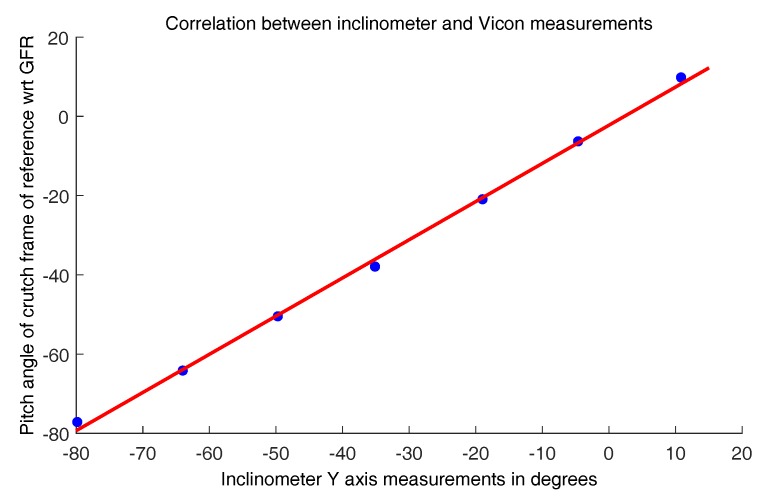
Best fitting straight line for the inclinometer measurements.

The calibration of the inclinometer was done with a 3D vision Vicon motion capture system (MCS) and its Nexus software, using 5 Bonita 10 cameras (one megapixel, 250 Hz) and a Vero camera (2.2 megapixels, 250 Hz). Four markers were stuck to a case parallel to the YZ plane of the crutch, and their 3D positions were captured by the MCS at a frequency of 100 frames per second. The orientation and pitch angle of the crutch was calculated from the positions of these markers. Seven experiments were performed with the MCS system to calibrate the inclinometer. In each experiment, the crutch was maintained with a constant orientation using a tripod with a graduated clamp. Between each two consecutive experiments, the crutch was rotated about 15 degrees with respect to its *y* axis (pitch angle). Figure 5 shows the correlation between the inclinometer measurements and the pitch angles calculated based on the markers’ positions measured by the MCS. As can be seen, a linear fitting can be used to establish a relationship between the inclinometer measurements and the pitch angles measured by the MCS. The best fitting straight line was obtained with $R^{2}=0.9983$ and a nonlinearity of 3.8°.

The inclinometer calibration results presented in Section 2.2 did not seem very good, compared with the results presented in [22], where the calculated nonlinearity of the static inclination measurement was 0.8°, while it was 3.8° for our crutch. Although the authors in [22] followed a different procedure, the typical nonlinearity according to the datasheet of the inclinometer used in our instrumented crutch was 1.2°, so the error that we obtained was probably due to small misalignments and measurement errors by the Vicon system when a marker was detected by various cameras, and due to occlusion by the bars of the tripod, one of the cameras was unable to detect it in another position. In fact, excluding the measurements at both extremes and at −35°, the nonlinearity was under 0.8° for our crutch.

### 2.3. Calibration of Angular Velocity Measurement

Finally, the angular velocity measurement calibration procedure will be detailed. The full-scale range of the gyroscope was set to ±500°/s (degrees per second (dps)), which covers the whole range of angular velocities during normal gait. The gyroscope was calibrated using a servomotor and following the procedure explained in [35].

**Figure 6:**
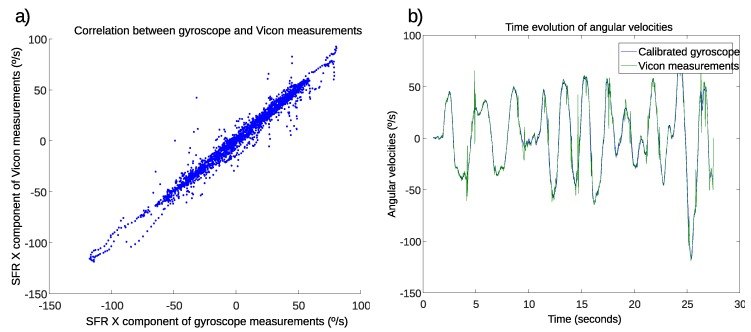
Validation of the measurements of the *x* component of the gyroscope. (**a**) Correlation between gyroscope and motion capture system (MCS) measurements. (**b**) Evolution of angular velocity measurements. SFR, sensors’ frame of reference.

In order to validate the calibration of the measurements in the *x* component of the gyroscope, which corresponds to the pitch angle of the crutch, another experiment was carried out using the Vicon MCS. In this experiment, the crutch was moved by hand back and forth without touching the ground, at different random angular velocities within the measurement range. The reference angular velocity was calculated based on the position of the markers attached to a case parallel to the YZ plane of the crutch. The *x* components of angular velocities were compared in the IMU sensors’ frame of reference (SFR). Figure 6a shows the correlation between gyroscope measurements and the angular velocities calculated using MCS measurements. The coefficient of determination of this cloud was $R^{2}=0.9721$. Figure 6b presents the time evolution of both the MCS signal, used as reference, and the calibrated gyroscope measurements.

The relatively low coefficient of determination and the existence of scattered points far from the thick linear cloud in Figure 6a were due to the impulses that can be seen in the green signal (Vicon signal) of Figure 6b. These impulses were perturbations in the MCS signal, which occurred after deriving an orientation signal with discontinuities. Hence, they did not represent the real angular velocity and should not be used as a reference. The orientation signal calculated based on Vicon markers’ positions had some discontinuities due to gaps and occlusion of some markers during some intervals. The effect of these discontinuities was observed in the angular velocity measurement even though the gaps were filled and the Vicon signal was filtered with a low-pass filter. Excluding these perturbations, it can be observed that the calibrated gyroscope measurements stuck very well to the MCS signal.

## 3. Algorithm for Estimating the Pitch Angle of the Crutch

The algorithm proposed in this section estimates the angle of rotation around the *y*-axis of the crutch (i.e., the pitch angle of the Tait–Bryan yaw-pitch-roll intrinsic angles). This angle is measured with respect to the plane perpendicular to the gravity vector. Hence, in the case of slopes, the angle was with respect to the plane perpendicular to gravity vector and not perpendicular to the slope. Note that although in the literature, several works defined this angle as the anteroposterior inclination of the crutch, this is not accurate, as the *x*-axis is not aligned with the direction of movement, so the angle of rotation around the *y*-axis is different from the angle of rotation around the frontal/coronal axis (i.e., anteroposterior angle), which is perpendicular to the longitudinal axis and sagittal axis of the patient. However, in the experiments performed to validate the algorithm, it was observed that the misalignment between the sagittal axis of the participants and the *x*-axis of the crutch was small, so the anteroposterior angle approximated the pitch angle of the crutch.

The measurements of the inclinometer and the gyroscope independently did not allow calculating an accurate estimation of the crutch pitch angle. For instance, the gyroscope suffered from the aforementioned drift effect when integrating its measurement, and the inclinometer was affected by nongravitational accelerations and impacts against the ground.

**Figure 7:**
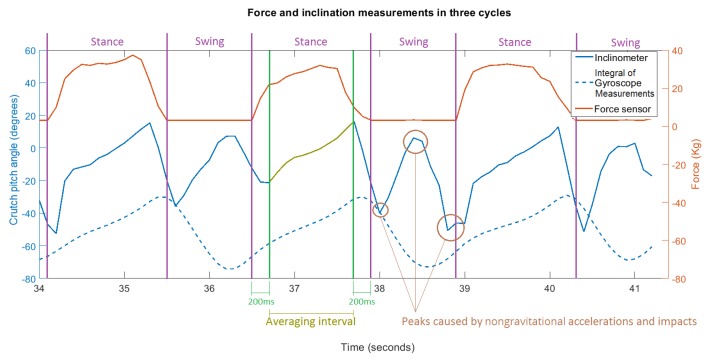
Force and inclination measurements during three cycles.

These effects can be seen in Figure 7, where the measurements of the force sensor (red), the inclinometer (continuous blue), and the integral of the gyroscope output (dashed blue) are illustrated for three crutch cycles. In addition, two different phases are highlighted: during the stance phase, the crutch tip was in contact with the ground, and the user was loading some weight on it, while during the swing phase, the force applied on the crutch was zero [22]. Note that the stance and swing phases throughout this article refer only to the crutch movement and not to the lower-limb kinematics of the user. As can be seen, the integral of the gyroscope measurement presented a significant drift in the form of an offset from the inclinometer signal, while the latter presented peaks when the tip hit the ground and higher accelerations were exerted. Note that negative inclination values indicated that the tip of the crutch was in front of the subject, while positive values meant that it was behind.

In order to calculate an accurate estimation of the pitch angle, in this section, a novel algorithm is proposed, which combines the data provided by the force sensor, the inclinometer, and the gyroscope. The fundamental idea of this algorithm is to integrate the data provided by the gyroscope to estimate the inclination angle, but correcting its drift or deviation periodically by using the data provided by the inclinometer in the middle of the stance phase (see yellow averaging interval in Figure 7), where nongravitational accelerations and angular accelerations are negligible. In addition, the algorithm assumes that the angular velocities around the global vertical axis are negligible. The drift compensation was carried out after each stance phase finished, using the sensor data stored during the last stance phase. Hence, it is important to detect stance and swing phases properly by using the force sensor signal.

**Figure 8:**
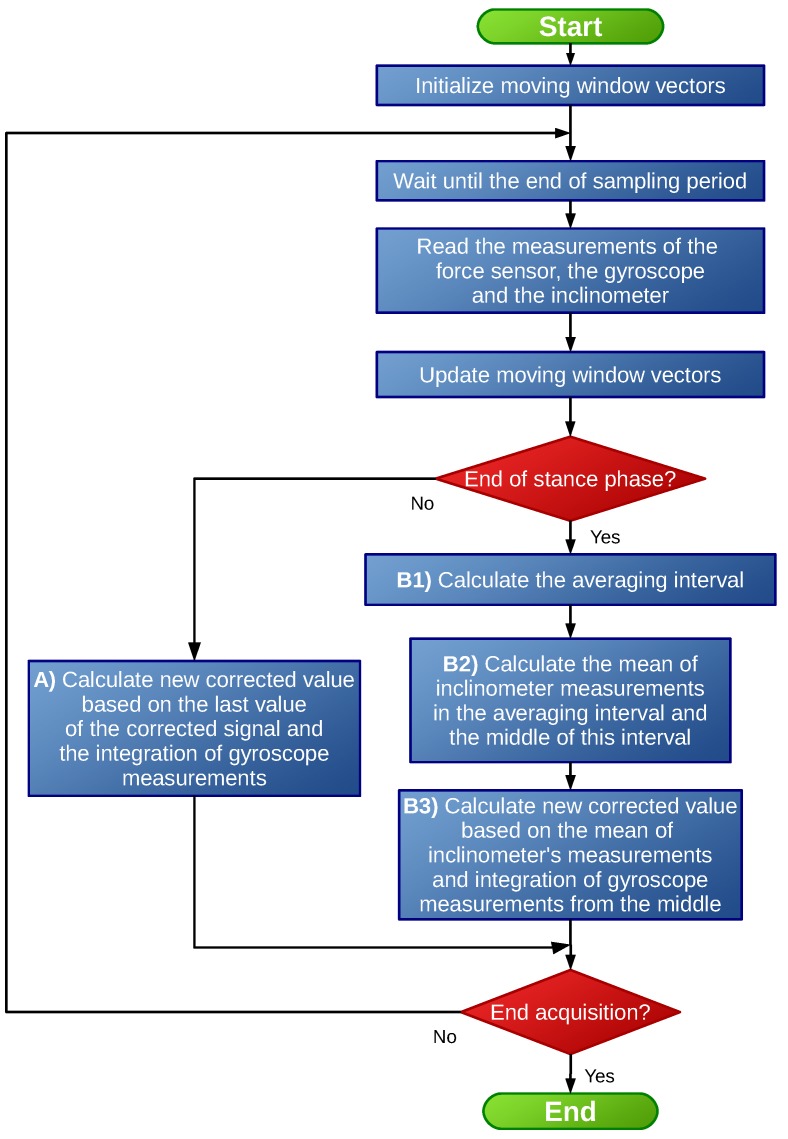
Block diagram of the algorithm used to estimate the pitch angle of the crutch.

The proposed algorithm is summarized in Figure 8. In order to implement the algorithm, the data associated with the previous stance phase were required. For that purpose, a properly-sized moving window was defined for the three inputs: the force provided by the force sensor, the angular velocity provided by the gyroscope, and the inclination angle measured by the inclinometer. This moving window was initialized at the beginning of the algorithm execution and then updated at each time step.

As described in the literature, the integration of the gyroscope signal generated a drift. In order to compensate this, the measurements provided by the inclinometer during the stance phase were used to update the estimated pitch angle value when the stance phase ended. The detection of the end of the stance phase was conducted using a simple minimum limit value $F_{min}$, as in the swing phase, the crutch was not in contact with the ground and no reaction forces existed. Hence, if the force sensor measured a force $f>F_{min}$, it was considered that the stance phase had begun. When the force fell under the limit value $f<F_{min}$, the end of this phase was detected, which corresponded to the start of the next swing phase. This value was established so that it was slightly higher than the limit of the dead zone and noise of the force sensor.

If the current time step did not correspond to the end of a stance phase (Path A in Figure 8), the estimated pitch angle was calculated by discrete integration of the angular velocity signal provided by the gyroscope, applying Heun’s integration method. Hence, for t=k,

(4) $$x_{k}=x_{k-1}+\frac{T_{s}}{2}\;\left(u_{k}+u_{k-1}\right)$$

where $x_{k}$ is the new value of the estimated pitch angle, $x_{k-1}$ is the previous estimated value, $T_{s}$ is the sampling period, and $u_{k}$ and $u_{k-1}$ are the last and second-to-last outputs (i.e., corresponding to the current and previous time steps) of the angular velocity provided by the gyroscope.

**Figure 9:**
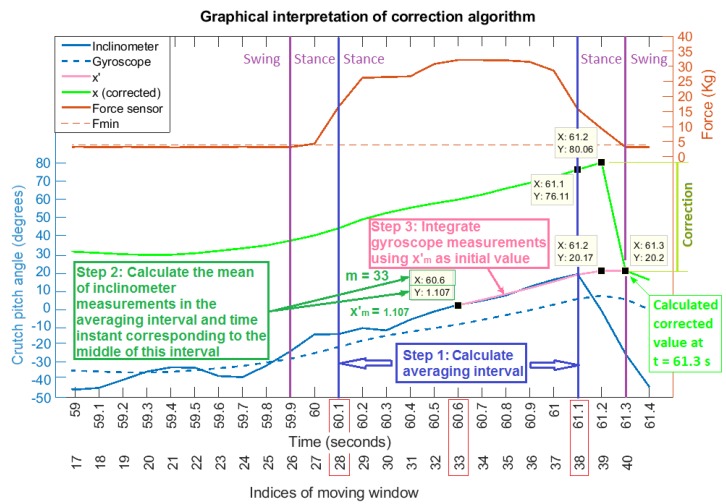
Graphical interpretation of the correction at the end of the stance phase. The continuous blue signal corresponds to the inclinometer’s measurements, the dashed blue signal to the integral of gyroscope measurements, the continuous red signal to the force measurements, the dashed red line to the selected force threshold, the light green signal to the outputs estimated by the algorithm at each time instant, and the pink signal to the past values estimated by the algorithm after applying the correction at the end of the stance phase. The plotted interval corresponds to a part of the first cycle after a turn.

Once the stance phase finished at t=k, the estimated pitch angle value was updated applying a three-step correction (Path B in Figure 8), as shown in Figure 9:

1. From the data stored in the moving window, the central interval of the data associated with the last stance phase was detected using the $F_{min}$ limit value nongravitational accelerations. In order to reduce the effect of possible phase change dynamics, the first and last values of this interval were neglected, obtaining the averaging interval as seen in Figure 9.
2. The algorithm calculated the mean $x_{m}^{\prime }$ of the inclinometer’s measurements within the defined averaging interval. In addition, the time step m<k associated with the middle of this interval was calculated.
3. An updated pitch angle value $x_{k}^{\prime }$ was estimated by integrating the gyroscope signal $u_{t}$ from t=m+1 to t=k and considering the inclinometer’s mean $x_{m}^{\prime }$ as the real value corresponding to t=m. This is,
  (5) $$x_{t}^{\prime }=x_{t-1}^{\prime }+\frac{T_{s}}{3}\;\left(u_{t-1}+u_{t}+u_{t+1}\right)\;t=m+1,m+2,\ldots ,k-1,k$$
  which was solved recursively using the past data stored in the moving window (when t=k, $u_{t}+u_{t+1}$ becomes $2\;u_{t}$). In this way, when t=k, the updated estimated pitch angle value $x_{k}^{\prime }$ can be calculated, and the output of the estimation algorithm was forced to this value $x_{k}=x_{k}^{\prime }$. Note that from this time step on, the algorithm continued to integrate the angular velocity signal (Equation (4)) (Path A in Figure 8).

Note that the proposed algorithm assumed that nongravitational accelerations were negligible in the middle of the stance phase. However, unlike the procedure followed by the authors in [21,22], this algorithm did not neglect nongravitational accelerations in the whole cycle, which allowed providing better accuracy.

## 4. Validation

### 4.1. Experimental Setup and Methods

In order to validate the proposed algorithm in a dynamic setting, a trial was conducted with three healthy volunteers in a room equipped with a Vicon MCS. The trials were approved by the Human Research Ethics Committee of the University of the Basque Country (UPV/EHU) with ethics Approval Code M10/2016/295MR1, and the subject characteristics are presented in Table 1.

Each individual was asked to walk with the instrumented crutch on their right hand, following a straight line at their normal and comfortable pace, trying to unload as much weight as possible from their left foot. Due to the size of the room and the limits of the capture volume of the MCS, the straight line was five meters long. Participants were asked to walk following the straight line, turn around, and come back to the initial position. Each participant completed this task twice.

The reference value for the pitch angle was measured using the aforementioned Vicon 3D motion capture system. This angle was obtained after calculating the orientation of the IMU frame of reference with respect to a global frame of reference. This involved calculating the orientation of the crutch reference frame based on the positions of the markers. The crutch reference frame was defined as: Z vector along the principal axis of the crutch, X along the handle, and Y accordingly, to create a right-handed reference system. In addition to the MCS frame of reference, which was defined using the Vicon wand, a global frame of reference (GFR) was defined as: Z vector pointing up and aligned with the gravity vector, X vector pointing north, and Y vector pointing west. This was done to calculate the misalignment between the crutch reference frame defined by the position of the markers and the IMU reference frame, following the method described in [36].

The Vicon MCS recorded the 3D position of the markers at a rate of 100 frames-per-second, while the acquisition device (myRIO) of the instrumented crutch registered the measurements from all the sensors at a frequency of 100 Hz. Synchronization between the MCS and the IMU was achieved by a fast impact of the crutch tip against the ground at the start of each experiment. This caused a peak in the Z component of the accelerometer when the crutch tip hit the ground, while the MCS measured the minimum height of the markers attached to the crutch when the impact occurred. The measurements of the MCS and the IMU were compared in the GFR.

The following tuning parameters were defined for the proposed pitch angle estimation algorithm. A moving time window of four seconds was defined to store the past data from the instrumented tip sensors. The limit force to detect the stance phase $F_{min}$ was selected to be 0.05 V (equivalent to 25.2 N). When calculating the central interval of the stance phase, the first and last 200 ms were not considered, as several effects due to the phase switch dynamics arose.

### 4.2. Results

**Figure 10:**
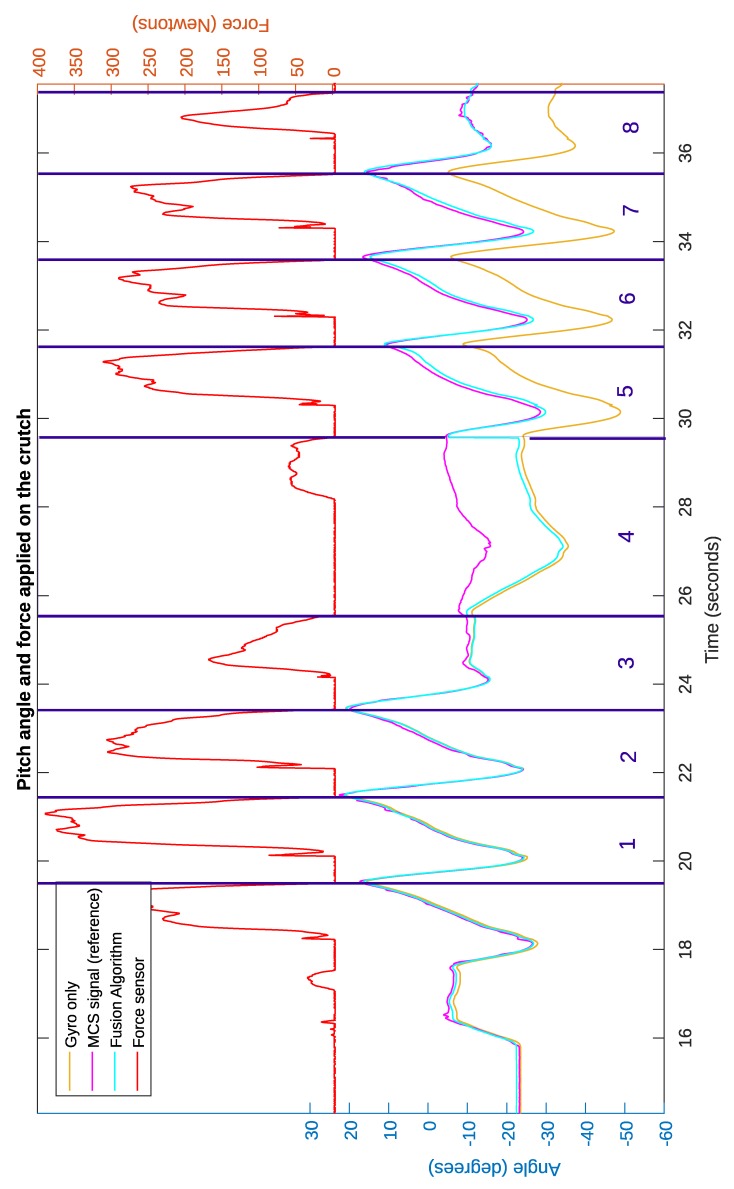
Estimated pitch angle obtained applying the presented algorithm.

Figure 10 depicts the time evolution of the input signals (force, gyroscope integration signal, and inclinometer measurements), the estimated pitch angle provided by the proposed algorithm, and the reference inclination measured by the 3D Vicon motion capturing system (MCS) for the data of the first task performed by the first subject. The vertical purple lines indicate the beginning and end of each cycle of the crutch movement. Each cycle started at the beginning of the swing phase and ended at the end of the next stance phase. The purple area was associated with the cycle, during which the subject turned around. As can be seen, the proposed algorithm (blue signal) provided an accurate estimation of the inclination if compared with the MCS measurement.

Note that the proposed algorithm updated the estimated value every cycle, when the stance phase ended. This is best seen at the end of the stance phase of the fourth cycle, after the subject turned around. It can be seen that, during this cycle, the estimated signal differed significantly from the reference, due to the error generated during the direction change. When the user turned around, the angular velocity on the global vertical axis was not negligible, and its projection on the *y* axis of the crutch caused a deviation from the reference pitch angle. However, at the end of the stance phase, based on the inclinometer’s measurements, the inclination angle estimation was updated to a value very close to the reference.

In addition, it can be seen that, in general, the estimated signal was slightly larger than the reference signal. This was due to noise in the inclinometer’s measurements and small nongravitational accelerations and angular accelerations during the stance phase. However, this deviation from the reference signal was small, as detailed in the next paragraphs.

Table 2 compares the root mean squared (RMS) errors obtained by integrating directly the gyroscope signal and applying the algorithm proposed in Section 3 with the three subjects. The cycles, during which participants turned, are highlighted in bold. SXRY means subject number X, repetition number Y. For example, Subject 1 completed eight cycles and turned on the fourth cycle in his first trial, as depicted in Figure 10, while in his second trial, he walked with longer strides and only completed five cycles.

As could be expected, the integral of the gyroscope was relatively good at the beginning, but it presented large errors in the long run due to turns and the drift effect. However, the signal estimated by the proposed algorithm showed small errors during the whole trial in all cases, except for the turning cycles. The average RMS error for all the cycles, excluding the cycles in which the subject was turning, was 1.13 degrees, and the maximum RMS error for one of these cycles was 2.75 degrees.

## 5. Discussion

The results in Section 4.2 showed that the assumptions listed in Section 3 led to a good approximation and that the described algorithm was able to correct the error of the gyroscope signal. Unlike most signal processing algorithms proposed in the literature for instrumented crutches, this algorithm did not assume that nongravitational accelerations due to quick movements during the swing phase and perturbations due to impacts with the ground were negligible.

Although the authors in [32] did not disclose the value of the mean RMS error in the estimation of the crutch inclination during normal walking, they presented a plot comparing the crutch tilt angles measured by an MCS and the approach proposed in that article. It can be easily appreciated from this plot that the mean RMS error must be over 5°, which is far from 1.13° of the algorithm proposed in this work. In addition, the authors in [33] compared the pitch estimation results obtained with different AHRSalgorithms based on IMU measurements for different tasks such as walking following a figure-eight path, stepping on a box, or climbing a staircase. Although these are more complicated tasks, the obtained RMS errors were over 2.7° for the figure-eight path and over 3.3° for stair climbing. Hence, based on the results obtained in the literature, the proposed approach provided good results.

Although the algorithm showed a good accuracy with the simple cases presented in this article, it cannot be generalized to the movements of a whole group of patients or potential users based on these data. The authors will conduct additional experiments in the future to test the algorithm with non-uniform motion conditions and more complicated tasks, both with healthy subjects and with patients.

In addition, the idea presented in this article was applied for estimating the pitch angle of the crutch, but the same concept might also work for estimating the 3D orientation of the crutch and the anteroposterior and mediolateral inclinations. The possibility of applying the approach only to a part of the stance phase instead of the whole central phase and the combination with other strategies (such as establishing a variation threshold in the module of the measurements of an accelerometer to ensure that nongravitational accelerations are negligible [37] or establishing a variation threshold in the module and direction of the gyroscope measurement to ensure linearity in the angular measurement) will be further analyzed in the future by the authors.

## 6. Conclusions

An instrumented crutch or cane for gait monitoring in rehabilitation can be used to monitor crutch movement variables, which might be related to the recovery status of a patient that requires rehabilitation. Estimating physical activity, performing clinical diagnosis, or improvement of gait training are some of the potential application areas of instrumented crutches. However, measuring the inclination of a crutch using only MEMS sensors involves tackling some challenges. For example, integrating the output of a gyroscope introduces a drift, which is hard to remove, and using accelerometers or inclinometers presents the drawback of nongravitational accelerations.

In this work, a new instrumented crutch tip was developed for measuring the axial forces and pitch angle of the crutch. After designing, manufacturing, and calibrating the prototype, a new algorithm for estimating the pitch angle of the crutch was implemented, based on the detection of crutch stance phases and the measurements of the inclinometer and gyroscope. The procedure was dynamically validated with three healthy volunteers, comparing the values estimated by the proposed algorithm and the data provided by a 3D motion capture system. The calculated average RMS error, excluding turning cycles, was 1.13 degrees, which validates the approach.

The presented results show that the algorithm is able to estimate the pitch angle of the crutch with good accuracy when the user is walking following a straight line. In addition, the algorithm corrects the deviation caused when the user turns or changes direction. This is a significant advancement, because it allows monitoring accurately and with a low computational cost an important crutch movement variable, which might provide additional objective and quantitative clinical information.

## Figures and Tables

**Table 1:** Characteristics of the subjects.

	Gender	Age	Weight (kg)	Height (cm)	Distance from Greater Trochanter to Floor (cm)
**Subject 1**	Male	26	77	182	90
**Subject 2**	Male	28	68	182	90
**Subject 3**	Male	23	66	170	87

**Table 2:** RMS errors for the six trials (expressed in degrees). S, subject; R, repetition.

Trial	Method	Cycle Number								Average ^1^
		1	2	3	4	5	6	7	8	
S1R1	Gyroscope integral	1.25	1.17	1.19	***17.81***	20.92	21.45	22.20	20.97	12.737
	Proposed algorithm	0.55	1.02	1.30	***16.49***	2.07	1.72	1.94	1.03	1.375
S1R2	Gyroscope integral	1.67	2.22	***13.53***	20.10	19.78				10.943
	Proposed algorithm	1.56	1.95	***15.31***	1.20	1.89				1.651
S2R1	Gyroscope integral	1.98	2.25	2.12	***23.81***	30.93	29.80			13.415
	Proposed algorithm	1.42	1.87	1.68	***23.59***	2.75	1.16			1.776
S2R2	Gyroscope integral	4.12	3.69	4.44	4.72	***18.47***	25.41	24.83		11.202
	Proposed algorithm	0.40	1.26	1.84	1.64	***15.96***	1.19	0.78		1.185
S3R1	Gyroscope integral	2.07	2.17	3.48	***29.70***	35.87	35.14			15.743
	Proposed algorithm	0.85	0.65	1.71	***27.19***	0.74	1.35			1.059
S3R2	Gyroscope integral	1.09	1.59	2.43	***28.52***	36.12				10.308
	Proposed algorithm	1.11	1.49	1.98	***27.45***	1.35				1.484

^1^ Excluding turning cycles.
